# Effect of weight loss on knee joint synovitis over 48 months and mediation by subcutaneous fat around the knee: data from the Osteoarthritis Initiative

**DOI:** 10.1186/s12891-024-07397-y

**Published:** 2024-04-17

**Authors:** Maximilian T. Löffler, Chotigar Ngarmsrikam, Paula Giesler, Gabby B. Joseph, Zehra Akkaya, John A. Lynch, Nancy E. Lane, Michael Nevitt, Charles E. McCulloch, Thomas M. Link

**Affiliations:** 1 Department of Radiology and Biomedical Imaging, University of California, 185 Berry St, Suite 350, Lobby 6, San Francisco, CA 94143 USA; 2 Department of Diagnostic and Interventional Radiology, University Medical Center Freiburg, Freiburg, Germany; 3 Department of Diagnostic and Interventional Neuroradiology, School of Medicine, Klinikum Rechts Der Isar, Technical University of Munich, Munich, Germany; 4 Department of Radiology, Ankara University Faculty of Medicine, Ankara, Turkey; 5 Department of Medicine and Center for Musculoskeletal Health, University of California, Davis, Sacramento, CA USA; 6 Department of Epidemiology and Biostatistics, University of California, San Francisco, CA USA

**Keywords:** Osteoarthritis, Weight loss, Synovitis, Mediation analysis, Effusion, Hoffa’s fat pad

## Abstract

**Background:**

Obesity influences the development of osteoarthritis via low-grade inflammation. Progression of local inflammation (= synovitis) increased with weight gain in overweight and obese women compared to stable weight. Synovitis could be associated with subcutaneous fat (SCF) around the knee. Purpose of the study was to investigate the effect of weight loss on synovitis progression and to assess whether SCF around the knee mediates the relationship between weight loss and synovitis progression.

**Methods:**

We included 234 overweight and obese participants (body mass index [BMI] ≥ 25 kg/m^2^) from the Osteoarthritis Initiative (OAI) with > 10% weight loss (*n* = 117) or stable overweight (< ± 3% change, *n* = 117) over 48 months matched for age and sex. In magnetic resonance imaging (MRI) at baseline and 48 months, effusion-synovitis and Hoffa-synovitis using the MRI Osteoarthritis Knee Score (MOAKS) and average joint-adjacent SCF (ajSCF) were assessed. Odds-ratios (ORs) for synovitis progression over 48 months (≥ 1 score increase) were calculated in logistic regression models adjusting for age, sex, baseline BMI, Physical Activity Scale for the Elderly (PASE), and baseline SCF measurements. Mediation of the effect of weight loss on synovitis progression by local SCF change was assessed.

**Results:**

Odds for effusion-synovitis progression decreased with weight loss and ajSCF decrease (odds ratio [OR] = 0.61 and 0.56 per standard deviation [SD] change, 95% confidence interval [CI] 0.44, 0.83 and 0.40, 0.79, *p* = 0.002 and 0.001, respectively), whereas odds for Hoffa-synovitis progression increased with weight loss and ajSCF decrease (OR = 1.47 and 1.48, CI 1.05, 2.04 and 1.02, 2.13, *p* = 0.024 and 0.038, respectively). AjSCF decrease mediated 39% of the effect of weight loss on effusion-synovitis progression.

**Conclusions:**

Effusion-synovitis progression was slowed by weight loss and decrease in local subcutaneous fat. Hoffa-synovitis characterized by fluid in the infrapatellar fat pad increased at the same time, suggesting a decreasing fat pad rather than active synovitis. Decrease in local subcutaneous fat partially mediated the systemic effect of weight loss on synovitis.

## Background

Symptomatic knee osteoarthritis (OA) occurs in an estimated 14 million people in the US [1] and prevalence substantially increased over the last 20 years of the twentieth century, most likely independent of demographic changes in age and BMI [2]. Obesity is a modifiable risk factor for OA. Previous studies showed the effect of weight change on knee OA: Weight gain promoted [3] and weight loss slowed cartilage degeneration [4] in subjects with OA risk factors.

The factors mediating the systemic and local effects of adipose tissue on OA are not well understood. Historically, obesity has been implicated in the pathogenesis of OA due to biomechanical reasons, but current research highlights its effect on OA via altered metabolism and inflammation [5]. Paracrine signaling from fat, rather than body weight was identified as a mediator of joint degeneration in mice [6]. A clinical phenotype of OA related to the metabolic syndrome has been described that is characterized by obesity and chronic low-grade inflammation [7]. Investigations focus on inflammation in synovial tissue (= synovitis) because inflamed synovium produces excess proteolytic enzymes that cause cartilage breakdown and, in turn, amplify inflammation potentially leading to a vicious circle [8].

Non-contrast enhanced MRI is a method to evaluate synovitis in OA that has been used in clinical trials and observational studies [9]. Previous imaging studies using unenhanced MRI investigated the relationship between synovitis, obesity, and OA. Irrespective of excess weight, progression of knee degenerative changes were higher in subjects with sustained synovitis assessed by semi-quantitative MRI [10]. Imaging markers of synovitis were higher in obese and overweight compared to normal weight subjects and higher in cases of severe OA defined by imaging and pain scores [11]. Weight gain was associated with increased synovitis imaging markers in overweight and obese women [12]. Beyond systemic effects of obesity, local SCF around the knee and the infrapatellar fat pad (IPFP) located in the joint capsule is of interest as being involved in the pathophysiology of knee OA [13]. A previous study found that joint-adjacent SCF played a role in the development and progression of knee OA [14]. However, the effect of local SCF on synovitis is unknown.

The purpose of this study was to investigate how weight loss and changes in local SCF around the knee impact knee joint synovitis development over 48 months in obese and overweight participants of the OAI. We also investigated these effects in separate analyses for women and men. We hypothesized that local SCF mediates the effect of weight loss on synovitis development.

## Methods

### Participant selection

The OAI is a multi-center, longitudinal, observational study investigating biomarkers that relate to the onset and progression of OA in a cohort of 4796 subjects (https://nda.nih.gov/oai/). The OAI recruited two primary subcohorts, one with and one without symptomatic knee OA at baseline. Symptomatic knee OA was defined, similar to the American College of Radiology (ACR) classification for clinical knee OA [15], by having both of the following criteria in at least one native knee at baseline: Frequent knee symptoms (pain, aching, or stiffness in or around the knee on most days) in the past 12 months for at least one month during the past 12 months and radiographic tibiofemoral knee OA, defined as definite tibiofemoral osteophytes (equivalent to Kellgren and Lawrence [KL] grade 2) on the fixed flexion radiograph. Subject for this study were selected irrespective of membership in any cohort.

**Fig. 1:**
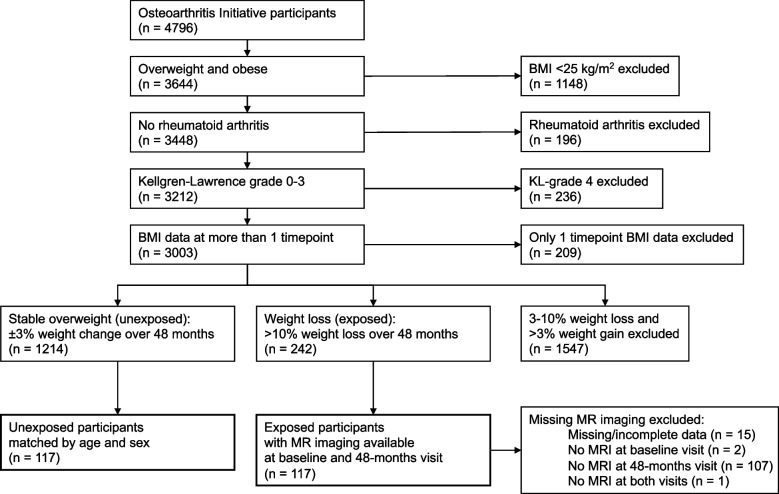
Subject selection

Overweight and obese participants (BMI ≥ 25 kg/m^2^) were selected from the OAI for an exposure-matched cohort study (Fig. 1). Participants without BMI data at more than one timepoint, with rheumatoid arthritis, and with advanced OA with a KL grade > 3 (representing end-stage OA) were excluded. Only participants who completed MRI at baseline and 48 months were included. Weight change over 48 months was calculated with linear regression models using annual BMI data in this period. Unexposed participants (stable overweight defined as <  ± 3% weight change) were individually matched to exposed participants (> 10% weight loss) by age (± 5 years) and sex to prevent association between exposure and the matching factors among participants at the start of follow-up. The PASE, a brief and reliable instrument for the assessment of physical activity, was collected for all selected participants at baseline [16].

### MR imaging

MR images were acquired on four identical 3 T scanners (Magnetom Trio; Siemens Healthineers, Erlangen, Germany) using quadrature transmit-receive coils (USA Instruments, Aurora, OH, USA). Three MRI acquisition sequences included in the OAI imaging protocol for the knee were used in this study [17]. Sagittal 3D dual-echo in steady state (DESS; TR/TE = 16.3/4.7 ms, flip angle = 25°) and coronal T1-weighted 3D fast low-angle shot (FLASH) water excitation (WE) sequences were used for subcutaneous fat measurements. Sagittal intermediate-weighted (IW) 2D turbo-spin echo (TSE) fat suppression (FS) and axial multiplanar reformation of 3D DESS sequences were used for synovitis readings.

### Semi-quantitative assessment of synovitis

MR images at baseline and 48 months were analyzed by three radiologists with 4 (MTL), 11 (CN), and 27 years of experience (TML). Images were read separately by two readers (MTL and CN). Final scores were adjudicated by the senior radiologist (TML) in case of mismatch. Images were reviewed in sequential order, unblinded to the time point of imaging visit, to increase sensitivity to change [18].

Two features of the MRI OsteoArthritis Knee Score (MOAKS) that are related to synovitis were semi-quantitatively assessed [19]. The MOAKS was employed in numerous previous studies [11, 12, 20–22] and is reliable and sensitive to change [23]. “Effusion-synovitis” represents a composite of synovial thickening and intraarticular joint fluid, as these cannot be distinguished on standard T2/IW/PD (proton density)-weighted images [24]. The amount of effusion-synovitis was evaluated in axial views of the 3D DESS sequence and graded as 0 = physiologic amount, 1 = small amount (fluid continuous in the retropatellar space), 2 = medium amount (with slight convexity of the suprapatellar bursa), and 3 = large amount (with signs of capsular distention). “Hoffa-synovitis” refers to diffuse hyperintense signal changes in T2/IW/PD fat-saturated images of the IPFP that are non-specific, but have been related to mild chronic synovitis [25]. The degree of hyperintensity in the IPFP was evaluated in mid-sagittal slices of sagittal T2 TSE FS. We added a reference to cartilage signal intensity to the original definitions of grades [19] as 0 = normal, 1 = mild (lower than cartilage signal), 2 = moderate (equal to cartilage signal), and 3 = severe hyperintensity (higher than cartilage or equal to fluid signal). Almost perfect inter-rater and intra-rater reliability for effusion-synovitis assessment (with weighted Cohen’s κ of 0.90 and 0.84, respectively) and almost perfect inter-rater and substantial intra-rater reliability for Hoffa-synovitis assessment (with weighted Cohen’s κ 0.93 and 0.76, respectively) were previously reported for different readers trained by the same expert radiologist as in this study [11].

### Subcutaneous fat measurements

**Fig. 2:**
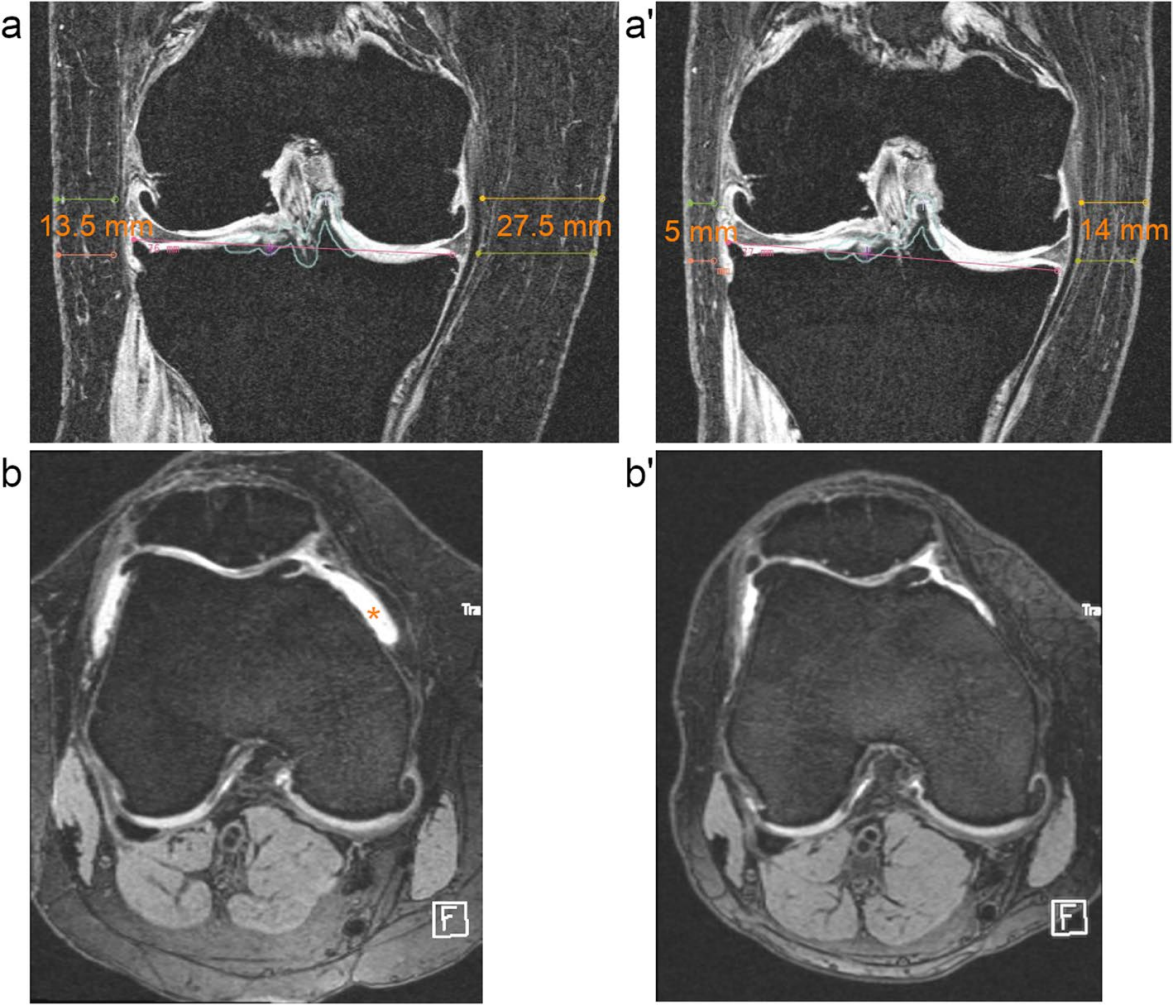
Subcutaneous fat (SCF) measurements and effusion-synovitis development. This 53-year-old woman had a baseline BMI of 35.2 kg/m.^2^ and lost 27.5% weight over 48 months. Medial and lateral SCF decreased by 13.5 mm and 8.5 mm, respectively, from baseline (**a**) to 48-months follow-up (a’). Effusion-synovitis (*) regressed from grade 2 at baseline (**b**) to grade 1 at 48-months follow-up (b’). Medial and lateral measurements were performed in coronal 3D FLASH sequences (a, a’) at two levels (at the tip of the medial tibial spine and at the level of the medial joint space) and averaged. Anterior measurements were performed in sagittal reformations (not shown). Semi-quantitative readings of effusion-synovitis by MOAKS were performed in non-enhanced axial reformations of 3D DESS sequences (b, b’)

SCF was measured anterior, medial, and lateral to the knee joint capsule in MRI at baseline and 48 months by two medical students who were trained by an experienced musculoskeletal radiologist. Medial and lateral measurements were performed in coronal 3D FLASH WE sequences (Fig. 2). Anterior measurements were performed perpendicularly anterior to the patellar tendon at half of its length in sagittal views of 3D DESS sequences [14]. The lateral and medial tibial spine served as anatomical landmarks to choose coronal and sagittal cross-sections, respectively, for reproducible measurements. The arithmetic mean was calculated as average joint-adjacent SCF (ajSCF). Good inter-rater and intra-rater reliability for SCF measurements were previously reported with coefficients of variation (CV) of 2.72% and 2.01%, respectively [14].

### Statistical analysis

Statistical analyses were performed using IBM SPSS Statistics 28 (IBM, Armonk, NY) with the extension PROCESS v4.2 for mediation analysis [26]. Level of statistical significance was defined as *p* < 0.05. Means of numeric variables (i.e., age, BMI, PASE, SCF measurements, synovitis scores) and proportions of categorical variables (i.e., sex, KL score) were compared between men and women using Student’s t-tests and chi-square tests, respectively. Means were compared using Welch’s instead of Student’s t-test when Levene’s test (*p* < 0.05) indicated that equality of variances could not be assumed. BMI, SCF measurements, and synovitis grades were compared between baseline and 48 months for groups with stable overweight and weight loss using paired t-tests.

To analyze associations between weight, SCF and synovitis change exposure-matched groups (stable overweight and weight loss) were coalesced into a single stratum for better efficiency. Change in synovitis scores between baseline and 48-month follow-up was dichotomized to reflect progression (change ≥ 1) or no progression (change < 1). First, we analyzed associations between baseline measures of BMI and ajSCF (= predictors) and effusion- and Hoffa-synovitis progression (= outcomes) in logistic regression models adjusting for age, sex, baseline PASE, and BMI as applicable. Then, we analyzed associations between weight loss and ajSCF change over 48 months (= predictors) and effusion- and Hoffa-synovitis progression (= outcomes) in logistic regression models adjusting for age, sex, baseline PASE, BMI, and SCF measurements. Odds ratios (ORs) and 95% confidence intervals (CIs) for synovitis progression were calculated. No formal correction for multiple testing was applied. Weight loss in percent BMI decrease and SCF change in mm decrease were transformed into SD as the unit of analysis for better comparability.

**Fig. 3:**
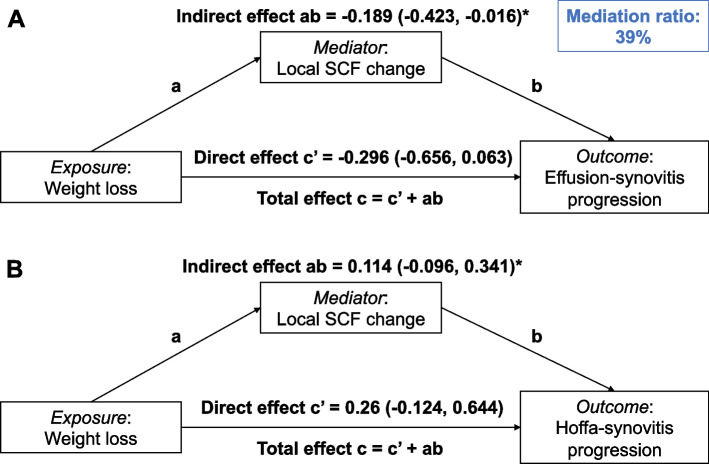
Direct acyclic graphs of mediation models. Beta coefficients (95% confidence intervals) are given for indirect and direct effects of weight loss on change of effusion-synovitis (**A**) and Hoffa-synovitis (**B**). All models are adjusted for age, sex, baseline BMI, PASE, and average joint-adjacent subcutaneous fat (ajSCF). * calculated as percentiles bootstrap confidence intervals

Mediation analysis assessed whether the effect of weight loss on synovitis progression was mediated by local SCF change when assuming that natural direct effect (path c’) and natural indirect effect (path ab) point in the same direction (Fig. 3). Outcomes for path ab and c’ are binary as in original analyses. Logistic regression models estimated the indirect effect of weight loss on synovitis progression through local SCF change and the direct effect of weight loss on synovitis through a process other than local SCF change adjusting for the same covariates as above. Percentile bootstrap confidence intervals were calculated for the indirect effect using 5,000 bootstrap samples [27]. The total effect (path c) was calculated as the sum of the direct and indirect effects log-odds ratios (= β coefficients). The proportion mediated was calculated as the indirect effect divided by the total effect ($ab/{(c}{\prime}+ab)$).

## Results

### Baseline characteristics

Baseline characteristics of both groups, weight loss and stable overweight, are shown in Table 1. The study cohort included 117 subjects with > 10% weight loss and 117 subjects with stable overweight over 48 months. Overall, there were 162 women and 72 men in the study cohort. Except for PASE, SCF measurements and KL grades there were no significant differences between men and women in baseline characteristics (all *p* > 0.05). PASE was significantly higher in men compared to women (170 ± 79 vs. 147 ± 73, *p* = 0.028). AjSCF, anterior, medial, and lateral SCF measurements were significantly higher in women compared to men (ajSCF: 14.6 ± 4.0 mm vs. 8.6 ± 2.6 mm, *p* < 0.001; anterior SCF: 7.4 ± 3.2 mm vs. 6.7 ± 1.6 mm, *p* = 0.027; medial SCF: 23.9 ± 7.3 mm vs. 13.0 ± 5.9 mm, *p* < 0.001; lateral SCF: 12.7 ± 4.8 mm vs. 5.9 ± 2.2 mm; *p* < 0.001). Distribution of KL grades was significantly different between men and women (*p* = 0.012) with significantly more KL grade 1 knees in men compared to women (33.3% vs. 19.8%; *p* = 0.025). There was no significant difference of synovitis grades between men and women (*p* = 0.960 for effusion-synovitis and *p* = 0.874 for Hoffa-synovitis).

**Table 1:** Baseline characteristics

	**Stable overweight**	** > 10% weight loss**
	*N* = 117	*N* = 117
**Women**	81 (69.2%)	81 (69.2%)
**Age, years**	61.0 ± 9.2	61.2 ± 9.3
**Body mass index, kg/m**^**2**^	30.4 ± 4.0	31.7 ± 4.1
**PASE**	158 ± 75	149 ± 76
**KL score**		
**0**	24 (20.5%)	28 (23.9%)
**1**	29 (24.8%)	27 (23.1%)
**2**	42 (35.9%)	41 (35.0%)
**3**	22 (18.8%)	21 (17.9%)
**SCF measurements**		
**AjSCF, mm**	12.4 ± 4.8	13.1 ± 4.3
**Anterior SCF, mm**	7.3 ± 2.8	7.1 ± 2.8
**Medial SCF, mm**	19.8 ± 8.6	21.4 ± 8.5
**Lateral SCF, mm**	10.5 ± 5.5	10.8 ± 5.0
**Effusion-synovitis grade**		
**0**	10 (8.5%)	10 (8.5%)
**1**	51 (43.6%)	49 (41.9%)
**2**	43 (36.8%)	49 (41.9%)
**3**	13 (11.1%)	9 (7.7%)
**Hoffa-synovitis grade**		
**0**	10 (8.5%)	8 (6.9%)
**1**	28 (23.9%)	33 (28.4%)
**2**	63 (53.8%)	54 (46.6%)
**3**	16 (13.7%)	21 (18.1%)

Parameters are showed as n (%) or mean ± SD

*PASE* Physical Activity Scale of the Elderly, *SCF* Subcutaneous fat, *ajSCF* Average joint-adjacent SCF. AjSCF is calculated as the arithmetic mean of anterior, medial, and lateral SCF

### Weight loss, subcutaneous fat change, and synovitis change over 48 months

BMI, SCF measurements, and synovitis grades at baseline and 48 months for both groups are shown in Table 2. Mean BMI significantly decreased by -4.6 ± 1.9 kg/m^2^ in subjects with weight loss (*p* < 0.001), whereas it slightly increased by 0.1 ± 0.6 kg/m^2^ in subjects with stable overweight over 48 months. At the same time, AjSCF significantly decreased by -1.7 ± 2.4 mm (12.6%) in subjects with weight loss (*p* < 0.001), whereas it significantly increased by 0.3 ± 1.7 mm (2.7%) in subject with stable overweight (*p* = 0.041; Fig. 2). Mean effusion-synovitis scores significantly increased by 0.2 ± 0.8 only in subjects with stable overweight (*p* = 0.002). In contrast, mean Hoffa-synovitis scores significantly decreased by 0.2 ± 0.5 only in subject with weight loss (*p* < 0.001).

**Table 2:** Change of parameters of 48 months

	**Stable overweight**				** > 10% weight loss**			
	**Baseline**	**48 months**	**Change**		**Baseline**	**48 months**	**Change**	
	**Mean ± SD**	**Mean ± SD**	**Mean ± SD**	***P*-value**	**Mean ± SD**	**Mean ± SD**	**Mean ± SD**	***P*-value**
**BMI, kg/m**^**2**^	30.3 ± 3.8	30.3 ± 3.9	0.1 ± 0.6	0.379	31.7 ± 4.1	27.2 ± 3.7	-4.6 ± 1.9	** < 0.001**
**AjSCF, mm**	12.4 ± 4.7	12.7 ± 4.5	0.3 ± 1.7	**0.041**	13.1 ± 4.3	11.4 ± 4.2	-1.7 ± 2.4	** < 0.001**
**Anterior SCF, mm**	7.3 ± 2.8	7.1 ± 2.6	-0.3 ± 1.5	0.067	7.2 ± 2.8	6.2 ± 2.3	-0.9 ± 1.7	** < 0.001**
**Medial SCF, mm**	19.8 ± 8.6	20.7 ± 8.2	1.0 ± 4.4	**0.021**	21.4 ± 8.5	18.5 ± 7.6	-2.8 ± 5.9	** < 0.001**
**Lateral SCF, mm**	10.4 ± 5.4	10.8 ± 5.7	0.4 ± 2.3	0.099	10.8 ± 5.0	9.6 ± 5.3	-1.2 ± 2.8	** < 0.001**
**Effusion-synovitis**	1.5 ± 0.8	1.7 ± 0.8	0.2 ± 0.8	**0.002**	1.5 ± 0.8	1.4 ± 0.8	0.04 ± 0.8	0.555
**Hoffa-Synovitis**	1.7 ± 0.8	1.8 ± 0.8	0.1 ± 0.5	0.162	1.8 ± 0.8	2.0 ± 0.8	0.2 ± 0.5	** < 0.001**

Effusion-synovitis relatively progressed from grade 1 to grade 2 in subjects with stable overweight from baseline to 48 months (grade 1/grade 2 frequency: 44/37% to 34/47%). Vice versa, effusion-synovitis relatively regressed from grade 2 to grade 1 in subjects with weight loss from baseline to 48 months (grade 1/grade 2 frequency: 42/42% to 52/31%). At the same time, Hoffa-synovitis showed a trend of increasing frequency of grade 3 in subjects with weight loss (18% to 29%).

Dichotomized change in synovitis-scores over 48 months showed similar differences between groups. Effusion-synovitis progressed significantly more frequently in subjects with stable overweight compared to weight loss (40.2 vs. 25.6%; *p* = 0.018). At the same time, Hoffa-synovitis progressed significantly less frequently in subjects with stable overweight compared to weight loss (12.0 vs. 25.9%; *p* = 0.007). Differences in frequencies remained significant after adjusting for age, sex, BMI, and PASE with 16.3% higher frequency for effusion-synovitis progression in subjects with stable overweight (CI: 4.2, 28.5%; *p* = 0.009) and -14.7% lower frequency for Hoffa-synovitis progression in subjects with weight loss (CI: -24.8, -4.5%; *p* = 0.005).

Prior to separate analyses of women and men we tested for sex-related interactions. There was no significant interaction of sex and weight loss on the effect of effusion-synovitis (*p* = 0.223) and Hoffa-synovitis progression (*p* = 0.758), respectively, or of sex and SCF decrease on the effect of effusion-synovitis (*p* = 0.191) and Hoffa-synovitis progression (*p* = 0.079), respectively. AjSCF significantly decreased in men by -1.4 ± 1.6 mm and in women by -1.8 ± 2.7 mm with weight loss. AjSCF significantly increased in men by 0.6 ± 0.9 mm, but not in women (0.2 ± 2.0 mm, *p* = 0.316) with stable overweight. In adjusted models, Hoffa-synovitis progressed significantly less frequently in women (-13.0%; CI: -25.7, -0.2%; *p* = 0.046) and men (-18.4%; CI: -35.4, -1.4%; *p* = 0.034) with stable overweight compared to weight loss.

### Associations between BMI or subcutaneous fat at baseline and synovitis progression

The odds of effusion-synovitis progression or Hoffa-synovitis progression over 48 months (dichotomized) did not significantly change for change in baseline BMI or baseline ajSCF, respectively (Table 3).

**Table 3:** Associations between baseline measures and synovitis progression

	**BMI, kg/m**^**2**^		**AjSCF, mm**	
	OR (95% CI)	*P*-value	OR (95% CI)	*P*-value
**Effusion-synovitis**	1.04 (0.97, 1.11)	0.321	1.08 (0.99, 1.18)	0.070
**Hoffa-synovitis**	1.00 (0.92, 1.08)	0.961	0.98 (0.89, 1.07)	0.658

Logistic regression models are adjusted for age, sex, PASE, and BMI as applicable

### Associations between weight loss or subcutaneous fat change and synovitis progression

The odds of effusion-synovitis progression significantly decreased, whereas the odds of Hoffa-synovitis progression significantly increased with weight loss (Table 4). Analyzing women and men separately, the association between weight loss and effusion-synovitis progression remained statistically significant in both sexes, but with larger effect size in men compared to women (Table 4). Weight loss was significantly associated with Hoffa-synovitis progression only in men (OR = 2.08; CI: 1.01, 4.29; *p* = 0.048), but not in women (OR = 1.33; CI: 0.90, 1.95; *p* = 0.149).

**Table 4:** Associations between weight loss or subcutaneous fat change and synovitis progression

	**Weight loss, SD**		**AjSCF decrease, SD**^**a**^	
	OR (95% CI)	*P*-value	OR (95% CI)	*P*-value
**All (*n* = 234)**				
**Effusion-synovitis**	0.61 (0.44, 0.83)	**0.002**	0.56 (0.40, 0.79)	**0.001**
**Hoffa-synovitis**	1.47 (1.05, 2.04)	**0.024**	1.48 (1.02, 2.13)	**0.038**
**Men (*n* = 72)**				
**Effusion-synovitis**	0.40 (0.20, 0.81)	**0.011**	0.15 (0.05, 0.50)	**0.002**
**Hoffa-synovitis**	2.08 (1.01, 4.29)	**0.048**	1.80 (0.64, 5.12)	0.267
**Women (*n* = 162)**				
**Effusion-synovitis**	0.69 (0.48, 0.98)	**0.039**	0.69 (0.49, 0.99)	**0.042**
**Hoffa-synovitis**	1.33 (0.90, 1.95)	0.149	1.47 (0.99, 2.19)	0.059

Logistic regression models are adjusted for age, sex, baseline BMI, PASE, and ajSCF. Statistically significant *p*-values are in bold

^a^ 5 missing average joint-adjacent subcutaneous fat measurements (AjSCF) due to missing MRI data (3 anterior SCF, 4 medial and lateral SCF measurements each)

**Fig. 4:**
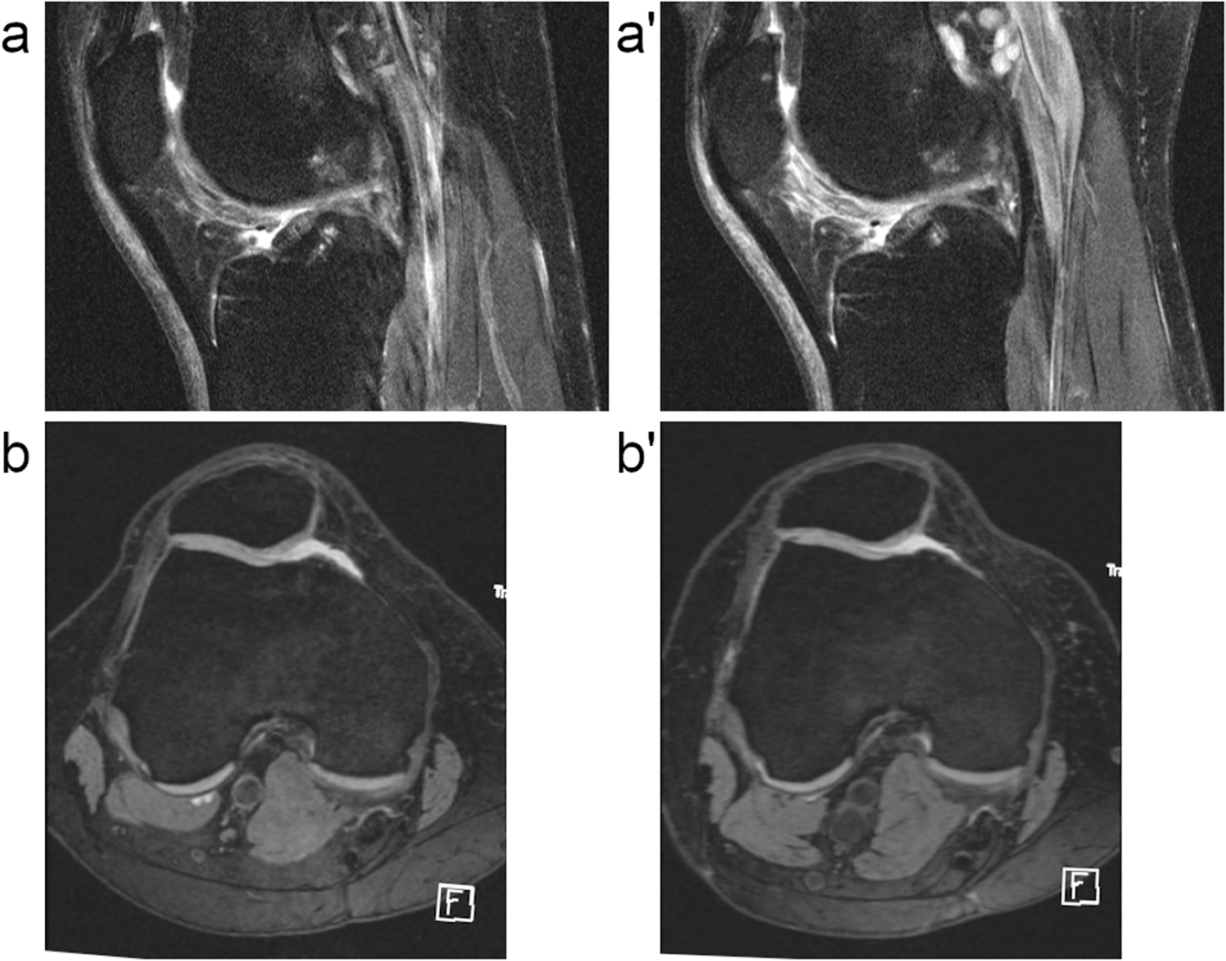
Development of Hoffa- and effusion-synovitis with weight loss. This 53-year-old women had a baseline BMI of 33.3 kg/m^2^ and lost 10.6% weight over 48 months. Medial and lateral subcutaneous fat (SCF) measurements decreased each by 5 mm from baseline to 48-months follow-up. Hyperintense signal changes in Hoffa’s fat pad anterior to trochlear cartilage increased from grade 1 at baseline (**a**) to grade 2 at 48-months follow-up (a’). Effusion-synovitis remained stable at grade 1 at baseline (**b**) and 48-months follow-up (b’) with MOAKS definitions not able to capture a slight decrease

The odds of effusion-synovitis progression significantly decreased, whereas the odds of Hoffa-synovitis progression significantly increased with decrease in ajSCF (Table 4). Analyzing women and men separately, the association between ajSCF decrease and effusion-synovitis progression remained statistically significant in both sexes. In contrast, decrease in ajSCF was not significantly associated with Hoffa-synovitis progression in women (OR = 1.47; CI: 0.99, 2.19; *p* = 0.059) and men (OR = 1.80; CI: 0.64, 5.12; *p* = 0.267). Representative cases of the associations between weight loss or SCF decrease and synovitis change are shown in Figs. 2 and 4.

### Mediation of the effect of weight loss on synovitis progression

Weight loss did not interact with the mediator, i.e. ajSCF change, in its effect on synovitis progression (*p* = 0.873). Natural direct effects and indirect effects of weight loss on progression of effusion- and Hoffa-synovitis by a pathway through ajSCF decrease are shown in Fig. 3. Weight loss had a significant negative indirect effect on effusion-synovitis progression mediated by ajSCF decrease (β = -0.189; CI: -0.423, -0.016). The positive indirect effect of weight loss on Hoffa-synovitis progression was not statistically significant (β = 0.114; CI: -0.096, 0.341). A mediation proportion of 39% was calculated for the indirect effects of weight loss through ajSCF decrease on effusion-synovitis progression.

## Discussion

Effusion- and Hoffa-synovitis represent two parameters to semi-quantitatively assess synovitis in non-enhanced knee MRIs [19, 28]. We found that weight loss and decrease in local SCF around the knee was associated with a decrease in effusion-synovitis, but with an increase in Hoffa-synovitis. This result is surprising given that both scores are supposed to evaluate synovial inflammation but may be explained by a shrinking Hoffa fat pad due to decrease in fat around the knee. Investigating the mediating effect of local SCF we found that 39% of the relationship between effusion-synovitis progression and weight loss was mediated by local SCF.

Obesity is a well-established risk factor for OA that affects joints beyond mechanical loading [29]. Although cartilage composition or thickness evaluated in MRI may be improved by weight loss in early knee OA, a recent systematic review concluded that current evidence is inconsistent in regard to specific structural effects of weight loss on imaging outcomes in overweight and obese persons [30].

Most risk factors for OA (age, obesity, trauma, and excess mechanical loading) are likely to influence OA pathogenesis by a pathway through synovial inflammation [31]. Synovitis might be present in early and advanced stage OA and involved in its development [8, 32–34]. In particular, effusion-synovitis and Hoffa-synovitis are strong predictors for the development of incident radiographic knee OA [35] and for pain progression [23]. Contrast-enhanced MRI is the reference for non-invasive assessment of synovitis in the knee joint [24] and showed moderate correlation with histologic macroscopic and microscopic synovial scores [36]. Nonenhanced MRI allows evaluation of effusion-synovitis [19, 37], but synovial proliferation cannot be distinguished from effusion since both appear hyperintense on fluid-sensitive sequences [24]. Furthermore, signal intensity alterations in the IPFP (= Hoffa-synovitis) in nonenhanced MRI are a sensitive but rather nonspecific finding that can represent synovial proliferation [28].

Interestingly, we found inverse associations of weight loss and SCF decrease with effusion- and Hoffa-synovitis progression, respectively. Previous studies described differences for relationships with effusion- and Hoffa-synovitis, too, but none of them specifically investigated individuals with weight loss. The presence of effusion-synovitis, but not Hoffa-synovitis (each grade ≥ 2) predicted cartilage lesions (semi-quantitative Whole-Organ MRI Score [WORMS]) at 30 months follow-up [33]. Comparing subgroups from the OAI incidence cohort (at least one knee with KL grade ≥ 2 at 48 months follow-up) with metabolic OA and OA related to physical activity Hoffa-synovitis was the only parameter that was significantly more prevalent in the active lean group that had baseline BMI < 25 kg/m^2^ [22]. Unfortunately, no information about weight change was provided in this study, but stable weight to moderate weight loss can be assumed in the active lean group given that they were required to have a PASE score ≥ 2 for strenuous sport/recreation activities. Furthermore, overweight significantly increased the odds of having effusion-synovitis – but not of having Hoffa-synovitis – two years prior to incident radiographic knee OA compared to normal weight [38]. A mediation study found that the effect of BMI on knee OA progression was mediated by effusion-synovitis progression, but not by Hoffa-synovitis progression [20].

Inconsistent with the slowing effect of weight loss on the progression of effusion-synovitis we found that weight loss promoted the progression of Hoffa-synovitis. Signal alterations in Hoffa’s fat pad (= IPFP) in unenhanced MRI are known to be a non-ideal surrogate for synovitis [28], due to its low specificity [21] and many diagnostic pitfalls (clefts, recesses, ganglion cysts, vessels) [39]. Moreover, the Hoffa-synovitis score showed lower reliability [23] and virtually no progression in weight gain [12] compared to effusion-synovitis. Hoffa-synovitis evaluates the IPFP, an intracapsular but extra-synovial structure that is enclosed by bone at two surfaces (femur and tibia). In healthy individuals, the volume of the IPFP increased with BMI showing a ceiling effect for those with BMI above 30 kg/m^2^ [40]. We should expect a negative linear correlation between weight loss and IPFP volume, as the mean baseline BMI of our study population was approximately at this level. Given the rigid, bony structures enclosing part of the IPFP we speculate that volume decreases of the IPFP due to weight loss could create spaces, eg in small fissures and clefts. These void spaces are filled by additional fluid increasing the T2/IW signal intensity. Another explanation could be that the decrease in fat in the region has reduced the cushioning effect of the fat and resulted in greater pressure on other soft tissue structures during ambulation such as synovium. Consistent with this hypothesis we found a longitudinal increase in signal intensity in the IPFP in individuals with weight loss. Consequently, we can speculate that our readings of Hoffa-synovitis progression were largely non-specific for synovial inflammation. This could add to the understanding insofar that Hoffa-synovitis readings are particularly non-specific in the context of weight loss, where signal increases are due to increasing fluid in a shrinking IPFP [40].

Previous studies have investigated the specific effect of adipose tissue on synovitis and knee OA assessed in unenhanced MRI. Progression of synovitis defined as the composite score of effusion- and Hoffa-synovitis had odds of 2.84 in women with weight gain compared to stable weight loss, but there were no significant association of synovitis progression with weight loss [12]. ORs for individual effusion- and Hoffa-synovitis score progression were not calculated. However, they reported progression rates of 6% and 9% for effusion-synovitis and of 1% and 0% for Hoffa-synovitis for the stable weight and weight loss groups, respectively. These rates are considerably lower than in our study likely due to a virtual absence of radiographic knee OA (93% vs. 46% with KL-grade 0 or 1) in their relatively healthy and young women population and a shorter follow-up interval (2.5 vs. 4 years). Furthermore, overweight and obese persons had a higher prevalence of effusion- and Hoffa-synovitis that correlated with cartilage composition and most features of semi-quantitative WORMS [11]. However, a recent study found no significant association between BMI categories and prevalence of effusion-synovitis or size of Hoffa-synovitis [20].

More than one third of the effect of weight loss on effusion-synovitis – but not on Hoffa-synovitis – was mediated by local SCF. In contrast to systemic low-grade inflammation caused by obesity and its effects on OA, the local effects of adipose tissue are largely studied in the context of the IPFP [5]. Only a few studies investigated SCF at the thigh, for example, in association with incident radiographic knee OA [41]. We evaluated local SCF adjacent to the knee joint. A previous study found significant cross-sectional and longitudinal association between joint-adjacent SCF (mostly lateral) and semi-quantitative and quantitative imaging markers of knee OA [14]. How local adipose tissue around the knee – other than the IPFP – affects synovial inflammation on a cellular and cytokine level and how this is different to systemic effects of adiposity could be a focus of future investigations. This study indicates partial mediation. The indirect effect on effusion-synovitis progression is statistically significant, yet the direct effect, despite being non-zero, has a confidence interval that overlaps with zero, which limits our capacity to establish complete mediation.

In sex-stratified analyses we found larger effect sizes for the association between weight loss or ajSCF decrease and effusion-synovitis progression in men compared to women and a significant association between weight loss or ajSCF decrease and Hoffa-synovitis progression only in men. Likely due to gynoid fat distribution, thickness of SCF around the knee at baseline was larger in women compared to men of the same mean BMI. However, absolute changes in local SCF did not significantly differ between men and women. Therefore, we can assume a disproportionate larger decrease in SCF in men compared to women that could have increased the effect on effusion-synovitis progression. Moreover, this could indicate that we observe a significant association with Hoffa-synovitis progression only in men due to relatively stronger volume decrease of the IPFP.

This study has limitations. First, the gold standard for identifying synovitis in imaging is contrast-enhanced MRI. Non-enhanced MRI cannot distinguish between effusion and synovial proliferation, though their occurrence is usually associated. Second, we considered this study exploratory and hypothesis forming. Third, our results are limited to overweight and obese persons (BMI >  = 25 kg/m^2^). Fourth, we did not include individuals with weight gain limiting the generalizability of the findings. Fifth, readers of MOAKS gradings were not blinded to timepoint. Sixth, we used a logistic regression analysis despite a rather small sample size. To address this issue we conducted a sensitivity analysis using Poisson regression to estimate risk ratios. This analysis confirmed the robustness of our primary logistic regression results except for the association between ajSCF decrease and Hoffa-synovitis that did not reach statistical significance but showed the same directionality. This consistency supports the reliability of our findings.

## Conclusions

We found that weight loss and reduction in subcutaneous fat around the knee decrease synovitis progression in overweight and obese persons. The systemic effect of weight loss on synovitis was partially mediated by local subcutaneous fat decrease. While effusion-synovitis in unenhanced MRI showed associations with weight loss in line with previous understanding, the imaging biomarker for synovitis of Hoffa’s fat pad should be used with caution in the context of weight loss because it could reflect effects that are non-specific for synovial inflammation.

## Data Availability

This article was prepared using a public-use data set of the Osteoarthritis Initiative (OAI) (https://nda.nih.gov/oai/).

## Abbreviations

ACR: American College of Radiology
AjSCF: Average joint-adjacent SCF
BMI: Body mass index
CV: Coefficients of variation
DESS: Dual-echo in steady state
FLASH: Fast low-angle shot
FS: Fat suppression
IPFP: Infrapatellar fat pad
IW: Intermediate-weighted
KL: Kellgren and Lawrence
MOAKS: MRI Osteoarthritis Knee Score
MRI: Magnetic resonance imaging
NIH: National Insittutes of Health
NIAMS: National Institute of Arthritis and Musculoskeletal and Skin Diseases
OA: Osteoarthritis
OAI: Osteoarthritis Initiative
OR: Odds ratio
OSMB: Observational Study Monitoring Board
PASE: Physical Activity Scale for the Elderly
PD: Proton density
SCF: Subcutaneous fat
TSE: Turbo-spin echo
WE: Water excitation
WORMS: Whole-Organ MRI Score
